# Ferritin and Encapsulin Nanoparticles as Effective Vaccine Delivery Systems: Boosting the Immunogenicity of the African Swine Fever Virus C129R Protein

**DOI:** 10.3390/v17040556

**Published:** 2025-04-11

**Authors:** Yue Zhang, Yi Ru, Longhe Zhao, Rongzeng Hao, Yang Yang, Yajun Li, Rong Zhang, Chenghui Jiang, Haixue Zheng

**Affiliations:** 1 State Key Laboratory for Animal Disease Control and Prevention, Lanzhou Veterinary Research Institute, College of Veterinary Medicine, Lanzhou University, Chinese Academy of Agricultural Sciences, Lanzhou 730046, China; zhang_yue_1995@163.com (Y.Z.); ruyi@caas.cn (Y.R.); zhaolonghe1992@163.com (L.Z.); haorongzeng@caas.cn (R.H.); yangyang01@caas.cn (Y.Y.); liyajun073@163.com (Y.L.); 2 China Agricultural Vet Biological Science and Technology Co., Ltd., Lanzhou 730046, China; zhangrongcvm@sina.com (R.Z.); jch18215171914@163.com (C.J.); 3 Gansu Province Research Center for Basic Disciplines of Pathogen Biology, Lanzhou 730046, China

**Keywords:** nanoparticles, immunogenicity, ferritin, encapsulin, African swine fever virus, targeted lymph nodes, C129R protein

## Abstract

Vaccination remains the most effective strategy for preventing infectious diseases. Subunit vaccines, which consist of antigenic components derived from pathogens, offer significant advantages in terms of biosafety, ease of preparation, and scalability. However, subunit vaccines often exhibit lower immunogenicity than whole-pathogen vaccines do. To address this limitation, coupling antigens with nanoparticles has emerged as a promising strategy for enhancing immune responses by mimicking pathogen structures and improving antigen presentation. This study evaluated the stability of ferritin (F-nps) and encapsulin (E-nps) nanoparticles and their efficient uptake by bone-marrow-derived dendritic cells (BMDCs) in vitro. In vivo studies demonstrated their effective targeting of lymph nodes. The African swine fever virus C129R protein was conjugated to ferritin and encapsulin nanoparticles to assess its ability to enhance antigen-specific immune responses. In murine models, both F-nps and E-nps significantly increased the immunogenicity of the C129R antigen, highlighting their potential as effective vaccine delivery systems. These findings underscore the promise of ferritin and encapsulin nanoparticles as delivery platforms for enhancing antigen immunogenicity and pave the way for the development of nanoparticle-based vaccines.

## 1. Introduction

Subunit vaccines have become a cornerstone of modern vaccinology because of their excellent biosafety profile and ease of preparation [1]. However, a significant challenge with subunit vaccines is their relatively low capacity to induce strong and long-lasting immune responses. A promising strategy for overcoming this limitation is the use of protein nanoparticles to present antigenic proteins. These nanoparticles mimic viral structures, enhancing antigen presentation and improving the activation of immune cells. By increasing the density of antigens and promoting their uptake by antigen-presenting cells (APCs), protein nanoparticles facilitate stronger B-cell activation and stimulate more robust immune responses [2]. This approach has proven effective in the development of vaccines for several infectious diseases, including influenza [3,4,5], SARS-CoV-2 [6,7,8], Zika [9], and HIV [10,11,12]. In these cases, protein nanoparticles have been shown to enhance immune responses, increase antigenicity, and improve vaccine efficacy. Their ability to mimic natural viral particles is critical for immune recognition, as their shape and size are optimized for APC uptake and immune activation [13,14]. In addition to their immunological advantages, protein nanoparticles offer several practical benefits for vaccine development. They can be synthesized rapidly and cost-effectively in engineered bacteria or cells, making them ideal for large-scale production. Furthermore, their inherent biocompatibility and safety make them attractive candidates for clinical use [15]. Consequently, protein-nanoparticle-based antigen presentation has emerged as a promising platform for the development of innovative vaccines [16].

A key advancement in this field is the use of the SpyTag/SpyCatcher system [17,18,19,20], which is derived from the fiber-binding protein FbaB of *Streptococcus pyogenes*. This system enables stable conjugation between nanoparticles and antigens, ensuring efficient antigen presentation and enhanced immunogenicity. By fusing SpyCatcher with nanoparticles and SpyTag with antigens [17,18,19,20,21], this system ensures the formation of stable nanoparticle–antigen complexes while preventing spatial obstructions that might affect assembly and stability. Studies have demonstrated that antigen-coupled nanoparticle scaffolds, such as those for hepatitis B, HIV, and SARS-CoV-2, exhibit significantly greater antigenicity than their unattached monomeric counterparts do [22,23,24]. Self-assembled protein nanoparticles, including ferritin [25,26,27] and encapsulin [28,29,30], have gained attention for their ability to form stable, well-defined structures capable of effectively activating immune responses.

This study compared ferritin (F-nps) and encapsulin (E-nps) nanoparticles in terms of their structure and stability, with the aim of identifying the two most promising candidates for further investigation of their in vivo metabolic distribution and immunogenicity. We used the African swine fever virus (ASFV) C129R protein as a model antigen. C129R is known for its immunomodulatory effects, including nuclease activity and the inhibition of type I interferon signaling [31,32]. The C129R-conjugated nanoparticle antigens were prepared and evaluated for the immune responses they elicit, providing valuable insights into the potential application of protein nanoparticle carriers in the development of vaccines for a wide range of infectious diseases.

## 2. Materials and Methods

### 2.1. Vector Design and Gene Synthesis

Two nanoparticle–protein vector systems were rationally engineered based on the structural frameworks established by Kanekiyo et al. [33] and Yin–Feng et al. [23]. The SpyCatcher (GenBank accession: MF974388.1) was genetically fused to the N-termini of ferritin subunits (GenBank accession: WP_011011871.1) and the C-terminus of encapsulin protomers (GenBank accession: WP_244261697.1). A (G_2_S)_3_ flexible peptide linker was employed to connect the nanoparticles to SpyCatcher, thereby promoting optimal particle folding and stability. The NdeI and XhoI restriction sites were chosen for gene insertion into the pET-28a plasmid, yielding the F-np and E-np constructs.

For antigen design, SpyTag was genetically fused to the N-terminus of enhanced green fluorescent protein (eGFP) and the C129R protein (GenBank No. QBH90550.1) using NdeI/XhoI-mediated directional cloning. The eGFP gene was subsequently inserted into the pET-28a plasmid, while the C129R antigen was inserted into pET-32a, resulting in the eGFP [34] and C129R constructs. All the recombinant plasmids were synthesized by Genewiz (Suzhou, China) and verified by sequencing.

### 2.2. Preparation of Nanoparticle Proteins and Conjugated Antigens

Expression plasmids encoding nanoparticle proteins and antigens were introduced into *Escherichia coli* BL21 (DE3) cells (Solarbio, Beijing, China) for colony screening in Luria–Bertani (LB) medium supplemented with kanamycin (or ampicillin). The cultures were then inoculated into 1 L of LB medium at a 1:100 dilution and incubated at 37 °C with shaking. When the optical density at 600 nm (OD600) reached 0.8, isopropyl β-D-1-thiogalactopyranoside (IPTG) was added at a final concentration of 1 mM to induce protein expression. After a 16 h induction period at 200 rpm, the cells were harvested by centrifugation at 8000 rpm for 30 min at 4 °C. The bacterial pellet was subsequently resuspended in equilibration buffer and subjected to sonication, after which the resulting supernatant was collected by high-speed centrifugation. Protein purification was performed using nickel affinity chromatography (GE Healthcare, Little Chalfont, UK). The purified proteins were analyzed by 12.5% sodium dodecyl sulfate–polyacrylamide gel electrophoresis (SDS–PAGE) and quantified via BCA.

Antigen-conjugated nanoparticles were synthesized by incubating excess antigenic proteins with ferritin or encapsulin in phosphate-buffered saline (PBS) at 4 °C overnight. The residual antigenic proteins were removed using a HiScreen Capto Core 700 device (Cytiva, Uppsala, Sweden), followed by SDS–PAGE analysis and quantification via BCA.

### 2.3. Nanoparticle Structure Characterization

Transmission electron microscopy (TEM) was employed to analyze the morphology of the protein nanoparticles. The nanoparticles were diluted to 0.2 mg/mL and drop-cast onto a 200-mesh carbon-coated copper grid for 2 min. The excess solution was carefully blotted dry with filter paper. The grid was then negatively stained with 2% phosphotungstic acid for 10 s, followed by blotting and air drying at room temperature. TEM imaging was conducted using an HT7700 microscope (Hitachi, Tokyo, Japan) operating at an acceleration voltage of 80 kV.

Dynamic light scattering (DLS) was employed to determine the size distribution/number and zeta potential of the protein nanoparticles. Prior to analysis, the purified protein samples were centrifuged at 12,000 rpm for 10 min to remove the aggregates. The supernatant was diluted to 0.2 mg/mL in PBS and transferred into disposable cuvettes. The hydrodynamic diameter/number and zeta potential were measured with a Malvern Zetasizer (Malvern Panalytical Ltd., Malvern, UK).

### 2.4. Nanoparticle Stability Studies

The stability of the purified protein nanoparticles was assessed under various conditions. The nanoparticles were dialyzed in PBS and then mixed with 20% (*v*/*v*) solutions of various reagents, including water, PBS, 10 mM dithiothreitol (DTT), 50 mM β-mercaptoethanol (β-ME), 50% glycerol, 500 mM glycine, 500 mM arginine, 500 mM sucrose, 1% Tween-20, and 10% polyethylene glycol 4000 (PEG4000). The mixtures were incubated at 4 °C for 2 weeks, followed by centrifugation at 12,000 rpm for 5 min to remove insoluble aggregates. The size distribution/number of the nanoparticles was measured using a particle size analyzer, and the remaining nanoparticle protein content was quantified by 12.5% SDS–PAGE.

To further evaluate the long-term storage stability, 100 mM arginine was added to both the F-np and E-np samples. The samples were stored at 4 °C, and their particle size/number was monitored at regular intervals (30, 60, 90, 120, and 150 days) using a particle size analyzer.

### 2.5. In Vivo Distribution of Nanoparticle Proteins

The in vivo distribution of nanoparticle proteins was studied using eGFP-based constructs. The purified eGFP particles and free eGFP were diluted to 1 mM. A total of 300 μL of each fluorescent protein mixture (eGFP, F-GFP, or E-GFP) was administered via subcutaneous injection into BALB/c mice, whereas a control group received 300 μL of PBS. The presence of fluorescent proteins at the injection site was monitored at 0, 12, 24, and 48 h postinjection. At 60 h postinjection, the tissues were harvested, and the distribution of the fluorescent proteins was analyzed using a small animal live imaging system (Guangzhou Biolight Biotechnology, Guangzhou, China). In addition, inguinal lymph nodes were collected, sectioned, and subjected to immunohistochemical (IHC) analysis. Follicular dendritic cells (FDCs) were specifically labeled with anti-CD35/CD21 antibodies to assess the localization of nanoparticle proteins within the lymph nodes.

### 2.6. Evaluation of Antigen Uptake In Vitro

Bone-marrow-derived dendritic cells (BMDCs) were isolated from the mice and cultured in RPMI 1640 medium supplemented with 10% fetal bovine serum (FBS; Gibco/Thermo Fisher Scientific Inc., Waltham, MA, USA), 20 ng/mL granulocyte–macrophage colony-stimulating factor (GM–CSF; Peprotech/Thermo Fisher Scientific Inc., Waltham, MA, USA), and 10 ng/mL interleukin-4 (IL-4; Peprotech). The medium was replaced every two days. After 7 days of culture, mature BMDCs were harvested, resuspended in RPMI 1640 medium, and seeded into 6-well plates at a density of 10^6^ cells per well. eGFP-labeled nanoparticles (F-GFP and E-GFP) and free eGFP were diluted to a final concentration of 100 μM. Next, 100 μL of eGFP, F-GFP, or E-GFP solution was added to each well, with 100 μL of PBS used as the control. After a 2 h incubation at 37 °C, a red lysosomal probe was added, and the cells were incubated for an additional 30 min. The cells were then washed 3–5 times with PBS to remove unbound fluorescent proteins. The uptake of fluorescent proteins by BMDCs was visualized and analyzed using laser scanning confocal microscopy.

### 2.7. Animal Vaccination

Forty female BALB/c mice were randomly assigned to four groups (n = 10 per group). Each mouse received a standardized dose of 40 μg of antigens (C129R, F-129R, E-129R) emulsified in Freund’s adjuvant at the same volume. The control groups were immunized with equal volumes of PBS emulsified in Freund’s adjuvant. For primary immunization, Freund’s complete adjuvant (FCA) was used, followed by booster immunizations with Freund’s incomplete adjuvant (FIA) every two weeks. Serum was collected weekly.

### 2.8. Antibody and Antibody Isotype Assay

An indirect ELISA was performed to assess the levels of specific IgG antibodies and the distribution of antibody isotypes in the serum samples. Briefly, 96-well plates were coated with 100 ng of C129R protein per well in carbonate buffer and incubated overnight at 4 °C. The plates were then blocked with 5% skim milk at 37 °C for 1 h, followed by washing with PBST and drying. Sera from immunized mice were serially diluted 1:1000 and added to the wells at 100 μL per well, followed by incubation at 37 °C for 1 h. The wells were then incubated with HRP-conjugated detection antibodies (goat anti-mouse IgG/IgG1/IgG2a) (ImmunoWay Biotechnology, Plano, TX, USA) diluted 1:5000 at 37 °C for 1 h. After incubation, TMB One Component HRP Microwell Substrate was added, and the reaction was allowed to proceed at room temperature for 15 min. The reaction was then stopped by adding 50 μL of 2 M H_2_SO_4_, and the absorbance at 450 nm was measured using an ELISA plate reader (Thermo Fisher Scientific, Waltham, MA, USA).

### 2.9. Flow Cytometry (FACS) and Analysis of Immune Cell Populations

Following booster immunization, the lymph nodes and spleens were harvested from the mice. Flow cytometry was conducted to evaluate the presence of follicular T helper (Tfh) cells, germinal center B (GCB) cells, activated B cells, and mature dendritic cells (DCs) in the lymph nodes, as well as the proportions of activated CD4+ and CD8+ T lymphocytes in the spleen. The fluorescence intensity of specific markers was analyzed using FlowJo software (Version X; TreeStar, Ashland, OR, USA). The following antibodies were used: anti-CD19 FITC, anti-PD-1 PE, anti-GL7 APC, anti-CD95 PE, anti-CXCR5 APC, anti-IgD APC, anti-CD3 FITC, anti-CD4 APC, and anti-CD8 PE (BioLegend, San Diego, CA, USA).

### 2.10. Cytokine Assay

Serum samples collected 28 days postimmunization were analyzed for IFN-γ and IL-4 concentrations using the Dakewe Cytokine Assay Kit (Dakewe Biotech Co., Beijing, China), according to the manufacturer’s instructions.

### 2.11. Safety Assessment

The safety and efficacy of vaccines are critical parameters in evaluating their overall performance. In this study, we assessed the safety of different vaccine formulations in BALB/c mice by monitoring body weight changes, analyzing blood biochemical indices, and evaluating pathological alterations in various organs. Body weight was recorded every two days over a 15-day period following antigen booster immunization. On day 35 postimmunization, anticoagulated blood was collected to measure biochemical indices. Additionally, major organs, including the heart, liver, spleen, lungs, and kidneys, were harvested for histopathological analysis. These organs were fixed, sectioned, and stained with hematoxylin and eosin (H&E) to evaluate any potential pathological changes.

### 2.12. Statistical Analysis

Data analysis and graphing were performed using GraphPad Prism 9.5 (La Jolla, CA, USA), with the data presented as the means ± standard errors of the means (SEMs). A *p* value < 0.05 was considered statistically significant.

## 3. Results

### 3.1. Design, Production, and Characterization of Protein Nanoparticles

**Figure 1:**
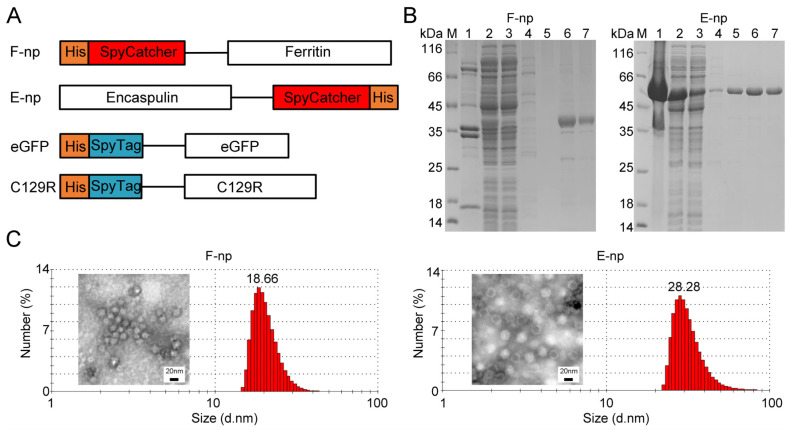
(**A**) Schematic illustration of the plasmid design for nanoparticle expression, including ferritin nanoparticles (F-nps) and encapsulin nanoparticles (E-nps), as well as the conjugated proteins eGFP and C129R. (**B**) SDS–PAGE analysis of F-np and E-np purification. The following samples were analyzed: precipitation after expression induction (lane 1), supernatants after expression induction (lane 2), flow-through samples (lane 3), and samples eluted with various concentrations of imidazole (80, 160, 300, and 500 mM; lanes 4–7). Lane M indicates the protein marker. (**C**) Structural characterization and particle size analysis of F-nps and E-nps using transmission electron microscopy (TEM) and dynamic light scattering (DLS).

Protein nanoparticles have garnered significant attention in vaccine research because of their capacity to enhance antigen presentation and immunogenicity, which ultimately improves immune protection. Their unique properties, such as the ability to mimic the natural structure of pathogens, enable better interaction with the immune system, making them ideal candidates for vaccine development. In this study, two protein nanoparticles—ferritin (F-nps) and encapsulin (E-nps)—were selected for their superior biocompatibility, stability, and ease of manipulation. The N-terminus of ferritin and the C-terminus of encapsulin were genetically engineered to be fused with SpyCatcher [19,23]. This allows the formation of stable conjugates with antigens. The SpyTag was attached to the N-terminus of eGFP and the ASFV C129R protein, facilitating the conjugation of antigens to the nanoparticles. Figure 1A provides a schematic illustration of the plasmid design for F-nps/E-nps and their conjugated antigens, eGFP and C129R. Plasmids encoding F-nps and E-nps were synthesized and expressed in *Escherichia coli* BL21 (DE3) cells. The successful purification of the nanoparticle constructs was confirmed by SDS–PAGE and Western blotting (Figure 1B), which demonstrated the purity and integrity of the proteins.

The formation of stable nanoparticles is essential for the efficacy of nanovaccines, as the particulate nature of these structures enhances antigen uptake by dendritic cells (DCs) and other immune cells. TEM was employed to examine the structural properties of the nanoparticles, revealing distinct morphological features (Figure 1C). Both F-nps and E-nps exhibited spherical shapes, with sizes ranging from 10 to 50 nm. This size range is ideal for efficient internalization by APCs, such as DCs, through receptor-mediated endocytosis [35]. DLS was used to further characterize the nanoparticles and confirm their nanoscale assembly. The DLS analysis corroborated the TEM findings, indicating that both F-nps and E-nps had narrow size distributions within the 10–50 nm range, which is ideal for enhancing cellular uptake and improving vaccine efficacy.

### 3.2. Evaluation of Nanoparticle Stability

**Figure 2:**
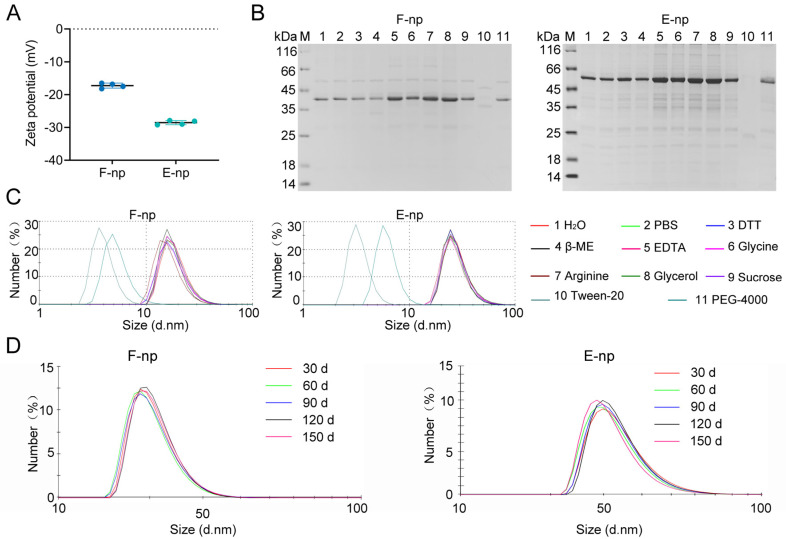
Stability study of the nanoparticles in various solutions. (**A**) Zeta potential measurements of ferritin (F-nps) and encapsulin (E-nps) nanoparticles were conducted to assess their colloidal stability. (**B**) Purified F-nps and E-nps were stored in PBS containing various 25% *v*/*v* solutions at 4 °C for two weeks. Following storage, the samples were centrifuged, and the resulting supernatants were analyzed via SDS–PAGE. The solutions tested included (1) water, (2) PBS, (3) 10 mM dithiothreitol (DTT), (4) 50 mM β-mercaptoethanol, (5) 50 mM EDTA, (6) 50% glycerol, (7) 500 mM glycine, (8) 500 mM arginine, (9) 500 mM sucrose, (10) 1% Tween-20, and (11) 10% PEG4000. (**C**) The particle sizes of the resulting protein aggregates were measured using DLS. (**D**) Measurement of the change in particle size of F-nps and E-nps over time following the addition of arginine. The samples were stored at 4 °C throughout the study.

Ferritin nanoparticles (F-nps) tend to aggregate and precipitate over time during storage, which may compromise their effectiveness as vaccine delivery systems. Zeta potential measurements revealed that F-nps exhibited lower zeta potential values than the other nanoparticle formulations did, suggesting a greater susceptibility to aggregation (Figure 2A). The zeta potential is an important indicator of colloidal stability, and its lower value in F-nps reflects a reduced repulsive force between particles, increasing the likelihood of particle aggregation and instability. To mitigate this instability and prevent aggregation, we further optimized the storage conditions by suspending the nanoparticles in PBS and testing various reagents, including reducing agents, amino acids, and dispersants, to identify the most effective stabilizers. Our results indicated that the addition of EDTA, glycine, arginine, and glycerol improved nanoparticle stability, as evidenced by the maintenance of consistent particle size and reduced aggregation (Figure 2B). Each of these agents likely interacts with the nanoparticle surface, either by providing steric stabilization or by chelating ions that could otherwise contribute to instability. Among the tested stabilizers, arginine was the most effective at enhancing the stability of both F-nps and E-nps. The incorporation of arginine significantly improved the long-term stability of both nanoparticle formulations, as they maintained their size and structural integrity for up to 5 months (Figure 2C). This level of stability is crucial for ensuring the reliability of nanoparticle formulations in vaccine development, as it ensures that the nanoparticles remain intact during storage, shipment, and preparation for immunization. The extended stability observed in the optimized formulations makes F-nps and E-nps suitable candidates for use in the development of nanoparticle-based vaccines.

### 3.3. In Vivo and In Vivo Distribution of the Nanoparticles

**Figure 3:**
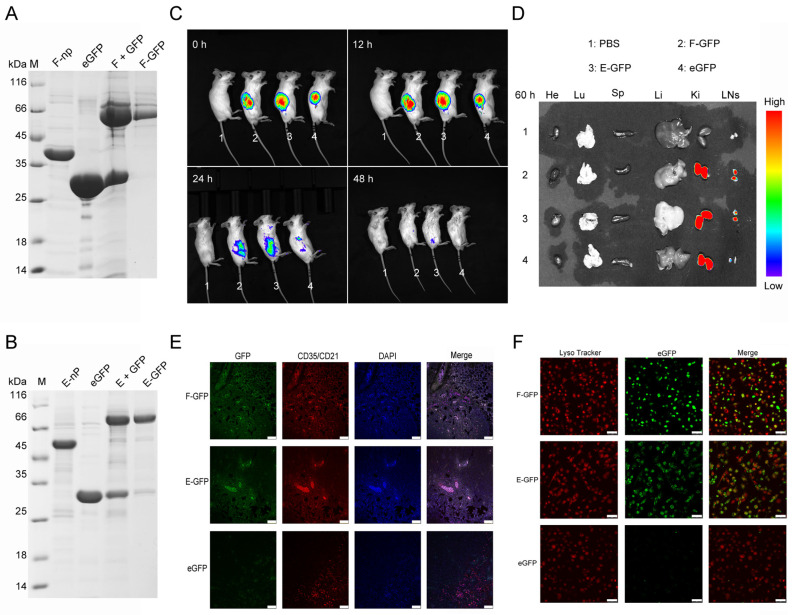
In vivo distribution and cellular uptake of the nanoparticles. (**A**,**B**) F-GFP and E-GFP nanoparticles conjugated with the eGFP protein were purified using molecular sieving and confirmed via SDS–PAGE analysis. (**C**) Fluorescence distribution of eGFP-labeled nanoparticles in the dorsal region of mice at 0, 12, 24, and 48 h following subcutaneous injection. (**D**) Imaging of lymph nodes and tissues from the heart (He), liver (Li), spleen (Sp), lungs (Lu), kidneys (Ki), and inguinal lymph nodes (LNs), which were isolated 60 h after the injection of eGFP and F/E-GFP nanoparticles, along with control group samples. (**E**) Lymph node sections labeled with CD35/CD21 antibodies were imaged using laser confocal microscopy to observe the distribution of fluorescent proteins (scale bar = 75 μm). (**F**) BMDCs were incubated with F/E-GFP nanoparticles or monomeric GFP for 2 h and then observed using confocal microscopy. Lysosomal compartments (eGFP and F/E-GFP, green) were labeled with LysoTracker (red). Scale bar = 50 μm.

To evaluate the in vivo distribution of F-nps and E-nps, both were conjugated with eGFP, yielding F-GFP and E-GFP, which were tracked following injection (Figure 3A–C). Within 48 h postinjection, fluorescence signals at the injection site were only faintly detectable, indicating efficient nanoparticle clearance (Figure 3C). Imaging of harvested tissues (spleen, heart, liver, lungs, kidneys, and lymph nodes) at 60 h postinjection revealed prominent fluorescence in the kidneys and inguinal lymph nodes (Figure 3D). The fluorescence intensity of the eGFP-labeled nanoparticles was significantly greater than that of the monomeric eGFP controls, confirming that the nanoparticles were trafficked to the lymph nodes, as previously reported [36,37,38]. Lymph nodes serve as crucial hubs for APCs, where they process antigens and initiate T- and B-cell immune responses. Tissue section analysis further demonstrated the colocalization of the nanoparticles with follicular dendritic cells (FDCs) within the lymph nodes (Figure 3E). To assess nanoparticle uptake, bone-marrow-derived dendritic cells (BMDCs)—key players in antigen presentation—were used. Confocal microscopy revealed rapid internalization of nanoparticle-conjugated fluorescent proteins by BMDCs, with colocalization in lysosomes observed within 2 h (Figure 3F). These findings suggest that nanoparticle conjugation enhances antigen delivery, facilitates cellular uptake, and contributes to subsequent immune activation. FDCs are known to retain nanoparticle-bound antigens through the complement pathway, thereby amplifying immune responses. Collectively, both F-GFP and E-GFP nanoparticles efficiently target immune organs, enhancing antigen reactivity and driving robust immune responses.

### 3.4. F/E-129R Nanoparticles Induce Strong Humoral and Cellular Immune Responses

**Figure 4:**
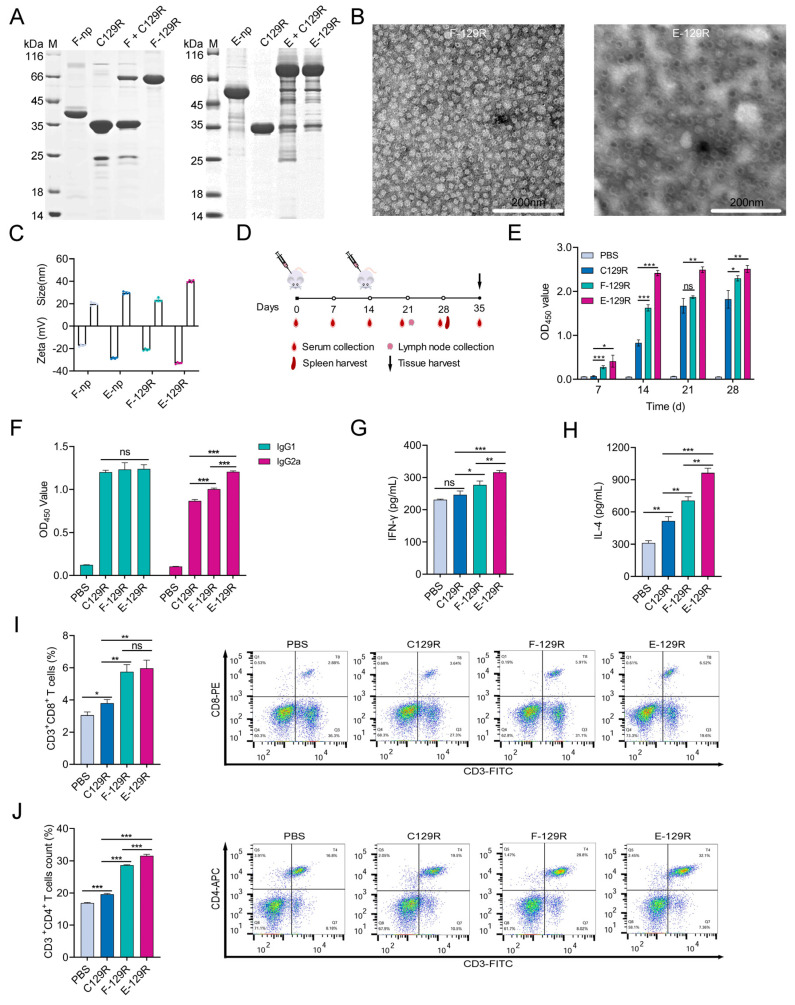
Preparation and immunological assessment of nanoparticles conjugated with the C129R protein. (**A**) F-129R and E-129R nanoparticles conjugated with the C129R protein were purified using molecular sieving and confirmed via SDS–PAGE analysis. (**B**) The structure of C129R protein-conjugated nanoparticles was observed by TEM (scale bar = 200 nm). (**C**) Particle size and zeta potential were analyzed using DLS before and after conjugation with the C129R protein. (**D**) Immunization flowchart depicting booster immunizations at two-week intervals, with each vaccine group receiving 40 μg of antigen per dose. (**E**) Changes in C129R-specific IgG levels were quantified by ELISA using serum samples collected at various time points at a 1:1000 dilution for optimal assay sensitivity. (**F**) Serum levels of C129R-specific IgG1 and IgG2a antibodies were quantified by ELISA on day 28 postimmunization via a 1:1000 dilution. (**G**,**H**) IFN-γ and IL-4 cytokine levels in serum samples collected on day 28 postimmunization were quantified. (**I**,**J**) The proportions of CD4+ and CD8+ T lymphocytes in the spleen were quantified by flow cytometry; ns: not significant, * *p* < 0.05, ** *p* < 0.01 and *** *p* < 0.001; unpaired *t*-test. The data are presented as the means ± SEMs of at least three biological replicates.

To assess the immunopotentiation of F-nps and E-nps against conjugated antigens, we conjugated them with the ASFV C129R protein. Following purification through molecular sieving (referred to as F/E-129R), the purity of the conjugated nanoparticles was confirmed via SDS–PAGE (Figure 4A). F-nps were fully consumed upon conjugation with C129R, showing nearly 100% conjugation efficiency, as no free F-np bands were detected. In contrast, some unreacted E-nps remained after conjugation, and after molecular sieving, the conjugation efficiency reached 87.8%, as determined by SDS-PAGE densitometric analysis. BCA assays quantified protein concentrations, ensuring a standardized 40 µg of total protein per dose, eliminating carrier-specific adjuvant effects. TEM images confirmed that both F-129R and E-129R nanoparticles formed spherical particles of uniform size and good homogeneity (Figure 4B). DLS measurements revealed minimal changes in particle size and zeta potential after conjugation with the C129R protein, indicating that the structural integrity of the nanoparticles was maintained. Moreover, the prepared nanoparticle antigens demonstrated high stability, further supporting their suitability for immunization applications (Figure 4C).

To assess the immunogenicity of the F/E-129R vaccine, BALB/c mice were immunized intramuscularly with an aluminum-adjuvanted vaccine containing a standardized total protein mass of 40 µg per dose. The mice were administered two doses biweekly, and serum samples, along with tissues, were collected at designated time points (Figure 4D). The ELISA results demonstrated that, compared with monomeric antigens, nanoparticle-based antigens induced significantly higher levels of specific antibodies (Figure 4E). For a comprehensive analysis of both humoral and cellular immune responses, we measured the IgG1 (Th2-associated) and IgG2a (Th1-associated) antibody levels in each group. Two weeks postimmunization, the IgG1 antibody levels were similar across the vaccine groups; however, the IgG2a levels were significantly elevated in the nanoparticle-conjugated groups, indicating enhanced cellular immunity (Figure 4F). These findings suggest that the nanoparticles preferentially stimulate Th1-type immune responses, which are crucial for cellular immunity. Cytokine production was also assessed to further investigate the immune response. The levels of IFN-γ (a Th1 cytokine) and IL-4 (a Th2 cytokine) were measured on day 28 postimmunization. Compared with the antigen-only group, the F/E-C129R group promoted the secretion of both cytokines, with significantly higher levels of IFN-γ and IL-4 compared to the antigen-only group (Figure 4G,H). These results demonstrate that the nanoparticle conjugates effectively stimulate both cellular and humoral immunity. Additionally, the proportions of CD4+ and CD8+ T lymphocytes in the F/E-129R groups were significantly higher than those in the monomeric vaccine group, further supporting the enhanced cellular immune response induced by the nanoparticle-conjugated antigens (Figure 4I,J). Overall, the results demonstrate that both F-nps and E-nps significantly enhance the immunogenicity of the C129R protein, promoting robust humoral and cellular immune responses. These findings underscore the potential of protein-nanoparticle-based platforms as effective vaccine delivery systems, capable of improving vaccine efficacy.

### 3.5. F/E-C129 Nanoparticles Promote Immune Activation in Lymph Nodes

**Figure 5:**
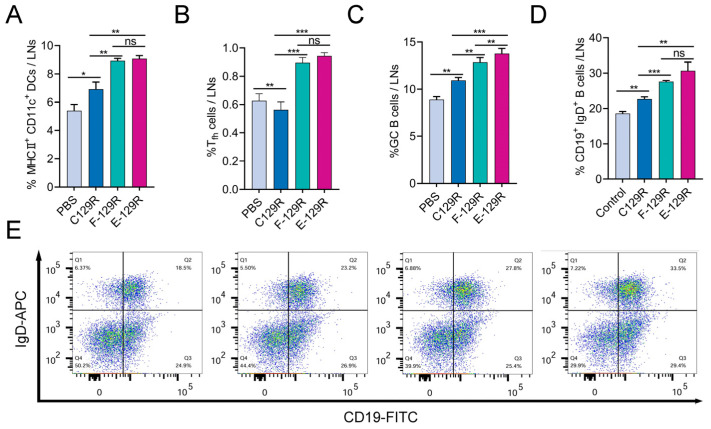
DC activation and synergistic T/B-cell responses in the lymph nodes. (**A**) DC activation in the lymph nodes was assessed by flow cytometry seven days after booster immunization. (**B**) The percentage of Tfh (CD4+CXCR5+PD-1+) cells in the lymph nodes was assessed by flow cytometry seven days after booster immunization. (**C**) The percentage of GCB (CD19+CD95+GL7+) cells in the lymph nodes was assessed by flow cytometry seven days after booster immunization. (**D**,**E**) B-cell activation (CD19+IgD+) was analyzed by flow cytometry seven days post booster immunization; * *p* < 0.05, ** *p* < 0.01, *** *p* < 0.001, ns: not significant; the results shown are the means and standard deviations of triplicate samples.

Lymph nodes are crucial sites for the activation of APCs, which process and present antigens, thereby initiating T- and B-cell responses [39]. Efficient antigen trafficking into lymph nodes plays a pivotal role in determining the strength and breadth of the immune response [40]. To assess the ability of F/E-C129 nanoparticles to stimulate immune activation in the lymph nodes, we analyzed inguinal lymph nodes from BALB/c mice 7 days after secondary immunization. Flow cytometry was employed to evaluate the activation of Tfh cells and DCs, both of which are critical players in immune activation. The results revealed that F/E-129R nanoparticles significantly enhanced the activation and proliferation of Tfh cells, and increased the expression of MHC-II, a marker of DC maturation (Figure 5A,B). These findings suggest that F/E-129R nanoparticles effectively promote the maturation of DCs, increasing their capacity to present antigens and activate T cells, which are essential for initiating a robust immune response.

In addition to the enhancement of T-cell responses, we further assessed the differentiation of GCB cells and the activation of B cells within the lymph nodes. Compared with the other groups, the F/E-129R nanoparticle group presented a substantial increase in the early maturation of B lymphocytes and GCB activation (Figure 5C,D,E). These findings indicate that F/E-129R nanoparticles not only augment the activation of Tfh cells but also stimulate B-cell responses, thereby enhancing the overall humoral immune response. This increase in the population of Tfh and GCB cells in the lymph nodes supports the notion that F-nps and E-nps, when used as vaccine carriers, can serve as effective adjuvants. By increasing dendritic cell activation and promoting more robust B-cell activation, these nanoparticles have the potential to enhance vaccine-induced immunity.

### 3.6. Safety Evaluation of F/E-129R Vaccines

**Figure 6:**
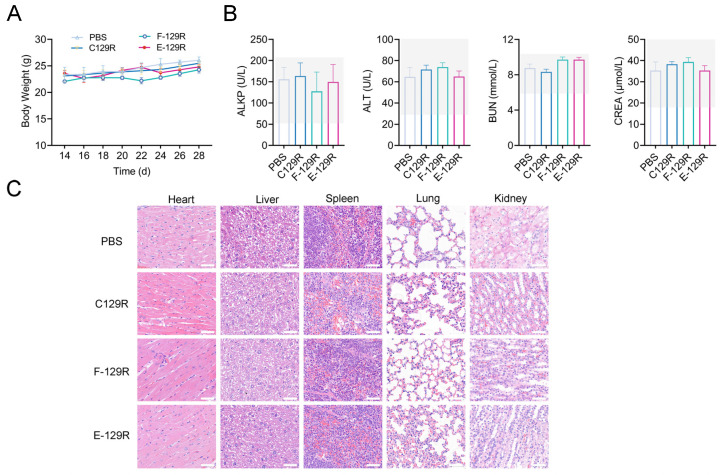
Safety assessment of F/E-129R vaccines. (**A**) Body weight changes in BALB/c mice over time following intramuscular injection of various vaccine formulations. (**B**) Serum levels of ALKP, ALT, BUN, and CREA were measured on day 35 postvaccination. The grey areas indicate the normal functional range for these markers. (**C**) Toxicity assessment of major organs (heart, liver, spleen, lungs, and kidneys) with H&E staining on day 35 postvaccination. Scale bar = 50 μm.

To assess the safety profile of F/E-129R nanoparticles, we carefully monitored body weight changes in BALB/c mice following vaccination at multiple time points. The data revealed no significant differences in body weight between the nanoparticle-treated group and the control group at any time point postimmunization, suggesting that the nanoparticles did not negatively impact the growth or development of the animals (Figure 6A). These findings indicate that the nanoparticles did not elicit any major adverse effects on general health or well-being. In addition to monitoring body weight, we assessed potential systemic toxicity by measuring several serum biomarkers indicative of organ function. These included alkaline phosphatase (ALKP), alanine aminotransferase (ALT), blood urea nitrogen (BUN), and creatinine (CREA) levels on day 28 postvaccination. The serum values for the F/E-129R nanoparticle group remained within the normal physiological range (Figure 6B), with no significant differences compared with those of the PBS-treated group.

To further evaluate the potential for organ-specific toxicity, we performed histopathological analysis on major organs, including the heart, liver, spleen, lungs, and kidneys, on day 28 postvaccination. Hematoxylin and eosin (H&E) staining revealed well-preserved tissue architecture in all organs of the F/E-129R nanoparticle group, with no signs of tissue necrosis or other pathological alterations. The tissue structure in the nanoparticle-treated group was comparable to that in the PBS-treated group (Figure 6C), providing further evidence of the biosafety of the nanoparticles. In conclusion, these results demonstrate that F/E-C129 nanoparticles do not induce significant toxicity or adverse effects in vivo. The absence of alterations in body weight, serum biomarkers, and organ histology supports the safety of these nanoparticles, confirming their suitability for use as vaccine carriers.

## 4. Discussion

Protein nanoparticles have emerged as promising vaccine carriers because of their ability to increase antigen immunogenicity through multivalent antigen display and efficient lymphatic targeting. This study systematically compared two self-assembling protein nanoparticles—ferritin (F-nps) and encapsulin (E-nps)—as platforms for delivering the ASFV C129R antigen. Our results demonstrate that both nanoparticle types significantly enhance humoral and cellular immune responses while maintaining excellent biocompatibility, underscoring their potential as next-generation vaccine carriers.

A key advantage of protein nanoparticles lies in their size-dependent effect on immune recognition and transport. The 20–50 nm size range of F-nps and E-nps falls within the optimal dimensions for lymphatic drainage (40 nm) [41], facilitating efficient transport to the lymph nodes. This size-dependent trafficking enables direct interaction with FDCs, which retain antigens via complement receptors, prolonging immune exposure and enhancing antigen presentation [33,34,35]. Notably, the spherical morphology and structural homogeneity observed via TEM likely contributed to enhanced APC uptake, as uniform particles are preferentially internalized by dendritic cells and macrophages [42,43]. Consequently, these particles are efficiently transported to the lymph nodes, triggering immune responses at these critical sites.

In murine models, both F-nps and E-nps significantly enhanced the immunogenicity of the C129R antigen, as evidenced by stronger T-cell and B-cell responses. The targeted delivery of these nanoparticles to the lymph nodes further corroborated their role in immune activation. Lymph nodes, which are central sites for immune responses, benefit from the targeted delivery of antigens to regions rich in immune cells, improving the efficiency of immune activation. Notably, DC activation is essential for effective antigen presentation and T-cell priming. In this study, F-129R and E-129R facilitated DC maturation, enhancing immune responses. Compared with traditional subunit antigens, nanoparticle-based vaccines are more efficiently captured and presented by DCs and macrophages, promoting early activation of both B cells and DCs and thereby fostering robust T-cell immune responses [41]. Both nanoparticle types were efficiently internalized by DCs, further amplifying immune activation.

This study provides valuable insights into the potential of protein-nanoparticle-based platforms for vaccine development. F-nps and E-nps offer significant advantages as vaccine delivery systems because of their stability, ability to enhance immunogenicity, and ease of large-scale production, making them ideal candidates for future vaccine platforms. Furthermore, the SpyTag/SpyCatcher binding system used in this study allows for efficient antigen coupling to nanoparticles, simplifying the vaccine preparation process. This system also offers versatility for rapid antigen replacement, making it adaptable for vaccines targeting a variety of pathogens [30,44]. Despite these promising results, several limitations must be considered. While murine models provide essential mechanistic insights into the behavior of these nanoparticles, further validation in swine—the natural host for ASFV—is crucial for assessing their clinical relevance. The long-term stability of these nanoparticles under fluctuating temperature conditions, a key consideration for field deployment, remains uncharacterized and warrants further investigation. Additionally, future studies should focus on evaluating the changes in immune memory cell subpopulations induced by nanoparticle-based vaccines and assessing the long-term persistence of the immune response to ensure the durability of vaccine-induced immunity.

In summary, this study systematically evaluated the preparation characteristics, stability, and in vivo distribution of ferritin and encapsulin nanoparticles as vaccine delivery platforms. Our findings confirm the significant advantages of these nanoparticles in enhancing the immunogenicity of the C129R antigen, providing a strong theoretical foundation for their continued exploration and application in the development of ASFV protein-nanoparticle-based vaccines.

## 5. Conclusions

This study underscores the potential of ferritin and encapsulin protein nanoparticles as effective vaccine carriers. We demonstrated the key properties of these nanoparticles, including their ability to be phagocytosed by BMDCs in vitro and their ability to enhance lymph node immune responses in vivo. Upon conjugation with the ASFV C129R protein, these nanoparticles were evaluated in immunization studies. Compared with immunization with the monomeric C129R protein, immunization with the nanoparticle-conjugated C129R protein significantly increased the proportions of Tfh and GCB cells in the lymph nodes. This enhancement resulted in stronger humoral and cellular immune responses. These findings highlight the potential of ferritin and encapsulin nanoparticles as effective carriers for enhancing immune responses to conjugated antigens in vivo. In conclusion, this study offers valuable insights into the selection and design of nanoparticle carriers for the development of future ASFV vaccines.

## Data Availability

Data are contained within the article.
